# Association of mutation profiles with metastasis in patients with non-small cell lung cancer

**DOI:** 10.3389/fonc.2024.1451576

**Published:** 2024-10-11

**Authors:** Yingxue Li, Zheng Zheng, Li Wang, Lin Han, Ying Du, Xuedong Zhang, Xia Liu, Jiaping Xie

**Affiliations:** ^1^ Department of Pathology, Liaocheng People’s Hospital, Liaocheng, Shandong, China; ^2^ Department of Pathology, School of Basic Medicine Science, Shandong University, Jinan, Shandong, China; ^3^ Department of Gynecology & Obstetrics, Liaocheng People’s Hospital, School of Medicine, Liaocheng University, Liaocheng, China; ^4^ Biomedical Laboratory, School of Medicine, Liaocheng University, Liaocheng, China; ^5^ Department of Gastroenterology, Liaocheng People’s Hospital, Liaocheng, Shandong, China; ^6^ Department of Gastroenterology, The Fifth People’s Hospital of Liaocheng, Liaocheng, Shandong, China

**Keywords:** non-small cell lung cancer, NSCLC, gene mutation, metastasis site, correlation analysis

## Abstract

**Objective:**

This study focused on the analysis of the correlation between common gene mutation types and metastatic sites in NSCLC patients.

**Methods:**

We retrospectively studied 1586 NSCLC patients and used fluorescence Polymerase chain reaction (PCR) to detect EGFR, ALK, ROS1, RET, MET, BRAF, HER2, KRAS, NRAS, and PIK3CA gene mutations, and also investigated sex, smoking status, age at diagnosis, histological type and TNM stage. In addition, we analyzed the site of metastasis in patients with stage IV NSCLC.

**Results:**

The EGFR-mutation group more frequently metastasized to lung (18.9%, P = 0.004), brain (18.9%, P = 0.001) and bone (27.1%, P = 0.004) than wild-type patients. ALK-mutation group (71.0%, P < 0.001), BRAF-mutation group (82.4%, P = 0.005) and NRAS-mutation group (100%, P = 0.025) were more likely to metastasize than the wild-type group. In the ALK mutation, lung metastasis (24.2%, P = 0.013), brain (24.2%, P = 0.007), bone metastasis (32.3%, P = 0.024), liver metastasis (19.4%, P = 0.001), and pleural metastasis (29.0%, P = 0.021) were common. In the KRAS-mutation group, lung metastasis (21.7%, P = 0.012) and brain metastasis (23.3%, P = 0.001) were more common. Less metastasis occurred in the HER2-mutation group (28.3%, P = 0.014). There was no difference in the RET, MET and PIK3CA mutations.

**Conclusion:**

Patients with ALK mutant, BRAF mutant or NRAS mutant were more prone to metastasis, while the HER 2 mutation group was less metastatic. Patients with EGFR mutant NSCLC are more likely to develop bone, lung, or brain metastasis.

## Introduction

Lung cancer is the most common cancer, and also the leading cause of cancer death in China (1). Non-small cell lung cancer (NSCLC) accounts for 75-80% of the total lung cancer cases, including adenocarcinoma, squamous cell carcinoma, adenosquamous carcinoma, large cell carcinoma, mucoepidermoid carcinoma, sarcomatoid carcinoma (2). Distant metastasis is the leading cause of most NSCLC deaths. Nearly one-third of patients with NSCLC have metastases at diagnosis, NSCLC often metastasizes to the brain, bone, pleural, liver and other organs (3–7).

The process of cancer metastasis and migration is a continuous process, where cancer cells can directly invade the surrounding organs/tissues, or invade the lymphatic system and vasculature and spread to other organs (8). Due to the complexity of the vascular and lymphatic systems, the site of metastasis is always unpredictable. One study showed that during tumor initiation, genomic instability causes various genetic mutations and leads to primary tumor cellular heterogeneity, thus increasing the possibility of tumor cell transformation, which is responsible for tumor spread and metastasis, such as immune evasion, epithelial-mesenchymal transition (EMT), and angiogenesis (9). Other studies have shown that the metastatic capacity of tumor cells is acquired through oncogene mutations in the initiation stage of early tumors and is stored in some subsets of tumor cells, known as “cancer stem cells” (10). Thus, somatic mutations seem to play a significant role in the tumor metastasis process in different cancer metastasis model studies.

With the rapid development of modern molecular biology techniques, more and more NSCLC driver mutations have been discovered, for example, epidermal growth factor receptor gene (EGFR), ananaplastic lymphoma kinase (ALK), rearrangements of the c-ros oncogene 1(ROS1), kirsten rat sarcoma viral oncogene homolog (KRAS), etc. Molecular genotyping has become critical in metastatic NSCLC, and the development of mutation-targeted therapies has revolutionized the treatment of NSCLC (11–14). With the continuous development of targeted therapy and immunotherapy, the overall prognosis of lung cancer is also constantly improving. To date, there is limited data on predicting or evaluating biological susceptibility factors at metastatic sites based on molecular profiles in NSCLC patients. A comprehensive analysis by Wu et al. found that both EGFR mutation and ALK mutations were associated with distant metastasis of NSCLC, however, no significant association was found between KRAS mutation and NSCLC metastasis (15). Several studies of ALK + NSCLC therapy have also shown that ALK + NSCLC has high rates of CNS metastasis even during first-line treatment with ALK TKI (16–19).

Based on these previous results, we propose that the biological predisposition to metastatic sites may differ between subsets of NSCLC molecules. There are no population-based data on the correlation of common gene mutation types with metastasis in NSCLC, especially the relationship between different gene mutation types and metastatic sites. This study focused on the analysis of the correlation between common gene mutation types and metastatic sites in NSCLC patients. These findings may provide a rationale for clinicians to develop immediate and accurate treatment plans for patients with lung cancer.

## Materials and methods

### Patients

A total of 1586 lung cancer patients who were treated in our hospital from December 2018 to August 2023 were enrolled in the study. This study was conducted with the approval of the Ethics Committee of Liaocheng People’s Hospital (NO. 2024086), and we guaranteed confidentiality throughout the study. Patient data were collected from our institutional cancer registry database and from patient follow-up visits to our outpatient office. The information collected from the records included patient characteristics, pathological diagnosis, metastasis site and follow-up data. Cancer staging was performed according to the Tumor node metastasis (TNM) classification of the International Union Against Cancer, 8th edition and histological typing was based on the World Health Organization classification.

### Reagents and gene mutation detection

DNA and RNA were extracted separately from paraffin-embedded lung cancer tissues using an FFPE DNA/RNA extraction kit (AmoyDx, Xiamen, China). Sample processing and DNA/RNA extraction were performed according to the manufacturer’s protocol. The EGFR, ALK, ROS1, rearranged during Transfection (RET), mesenchymal-epithelial transition factor (MET), v-raf murine sarcoma viral oncogene homolog B (BRAF), human epidermal growth factor receptor 2 (HER2), KRAS, neuroblastoma RAS viral oncogene homolog (NRAS), and phosphatidylinositol 3-kinase (PIK3CA) gene mutations were detected using the human lung cancer multi-gene mutation detection kit (fluorescence PCR method) (AmoyDx, Xiamen, China). Detailed Gene test sites as listed in **Supplementary Material**, **Table 1**. Amplification Refractory Mutation System-Polymerase Chain Reaction (ARMS-PCR) was conducted on a cobas Z480 platform (Roche, Switzerland), and the amplification program was set up as follows: Phase 1: 5 min at 42°C and 5 min at 95°C; Phase 2: 25 s at 95°C, 20 s at 64°C, 20 s at 72°C for 10 cycles; Phase 3: 25 s at 93°C, 35 s at 60°C, 20 s at 72°C for 36 cycles; signal collection: The FAM and VIC signals were collected at 60°C in phase 3.The experiment process was performed according to the product instructions.

**Table 1:** Basic clinical information of 1586 cases of patients with non-small cell lung cancer.

Clinical characteristics	Number of patients (%)
** Total**	1586
Age		
<70	1157 (73.0)
≥70	428 (27.0)
NA	1
Sex		
male	752 (47.4)
female	834 (52.6)
Smoking	
smoked	557 (35.1)
no-smoked	945 (59.6)
NA	84 (5.3)
Histology type	
Adenocarcinoma	1431 (90.2)
Squamous cell carcinoma	71 (4.5)
Adenosquamous carcinoma	21 (1.3)
Neuroendocrine carcinoma	11 (0.7)
Sarcomatoid carcinoma /sarcoma	9 (0.5)
Large cell carcinoma	1
Undifferentiated carcinoma	42 (2.6)
TMN	
I	421 (26.5)
II	72 (4.5)
III	198 (12.5)
IV	794 (50.1)
NA	101 (6.4)

NA, not available.

### Statistical analysis

Statistical analysis performed by IBM SPSS statistics 27.0. Enumeration data expressed as numbers, the association between different groups were analyzed by Chi-square test. When the chi-square test was not met, Fisher’s exact probability test was used. Statistical significance was set at P value less than 0.05.

## Results

### Patient characteristics

The clinicopathological characteristics of the 1586 patients are summarized in **Table 1**. Tissue types included 684 surgical resection specimens, 667 bronchoscopic biopsy or crude needle biopsy specimens and 194 pleural effusion or pericardial effusion specimens. Among the 1586 patients, 752(47.4%) were males and 834 (52.6%) were females, with an average age of 62.8 years (range = 23–92 years). Among the 1586 lung cancer cases, 84 had an unknown smoking history, 557 (35.1%) were smokers, and 945 (59.6%) were non-smokers. Tumor staging was performed according to the 8th edition of Lung cancer staging criteria for IASLC (international association for the study of lung cancer); 421 cases (36.5%), 72 cases (4.5%), 198 cases (12.5%) and 794 cases (50.1%) were in stages I, II, III, and IV, respectively, and 101 cases staging were unknown. The pathological types of these patients were mostly adenocarcinoma (1431 cases, 90.2%), squamous cell carcinoma (71 cases, 4.5%), adenosquamous carcinoma (21 cases, 1.3%), neuroendocrine carcinoma (11 cases, 0.7%), sarcomatoid carcinoma and sarcoma (8 cases, 0.5%), large cell carcinoma (1 cases, 0.06%), and undifferentiated carcinoma (42 case, 2.6%).

### Molecular characteristics

**Figure 1:**
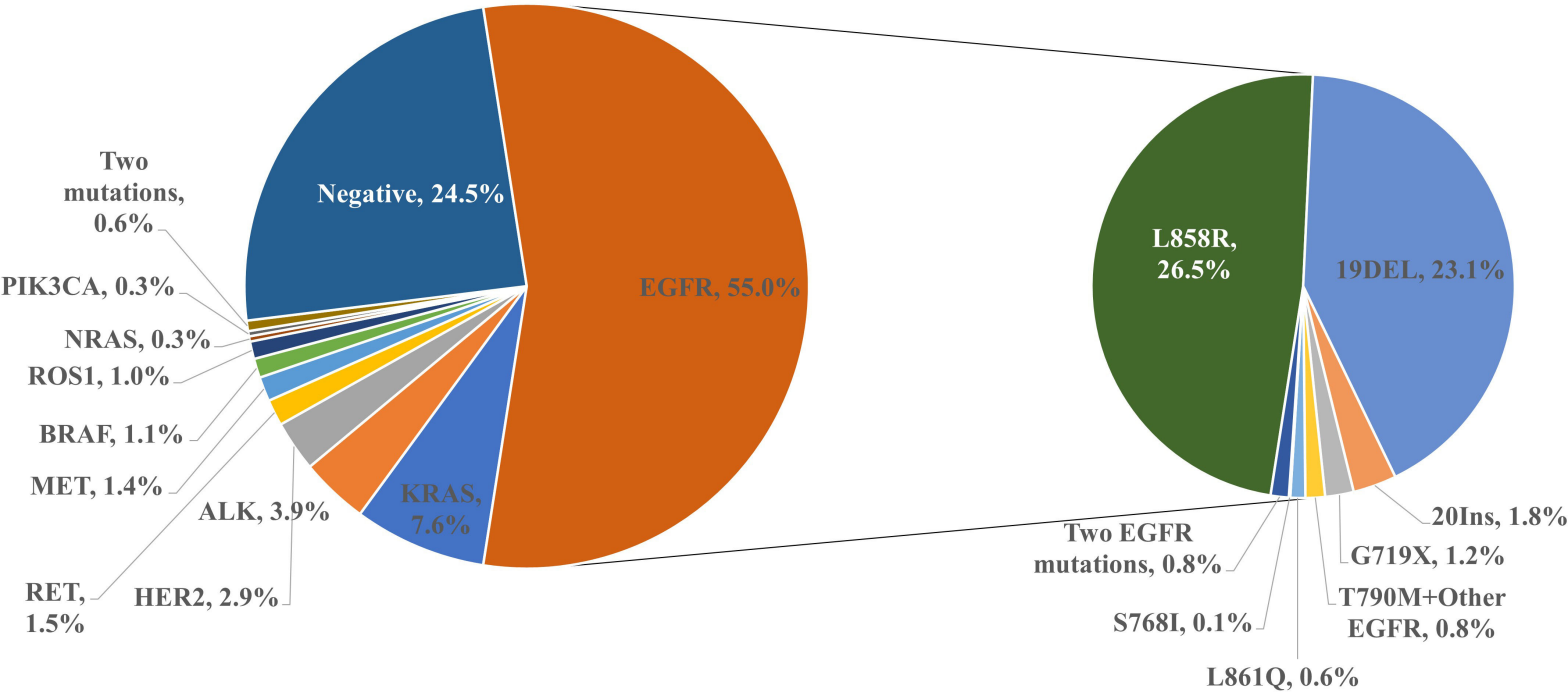
Frequency of detection of gene mutation in non-small cell lung cancer.

**Figure 2:**
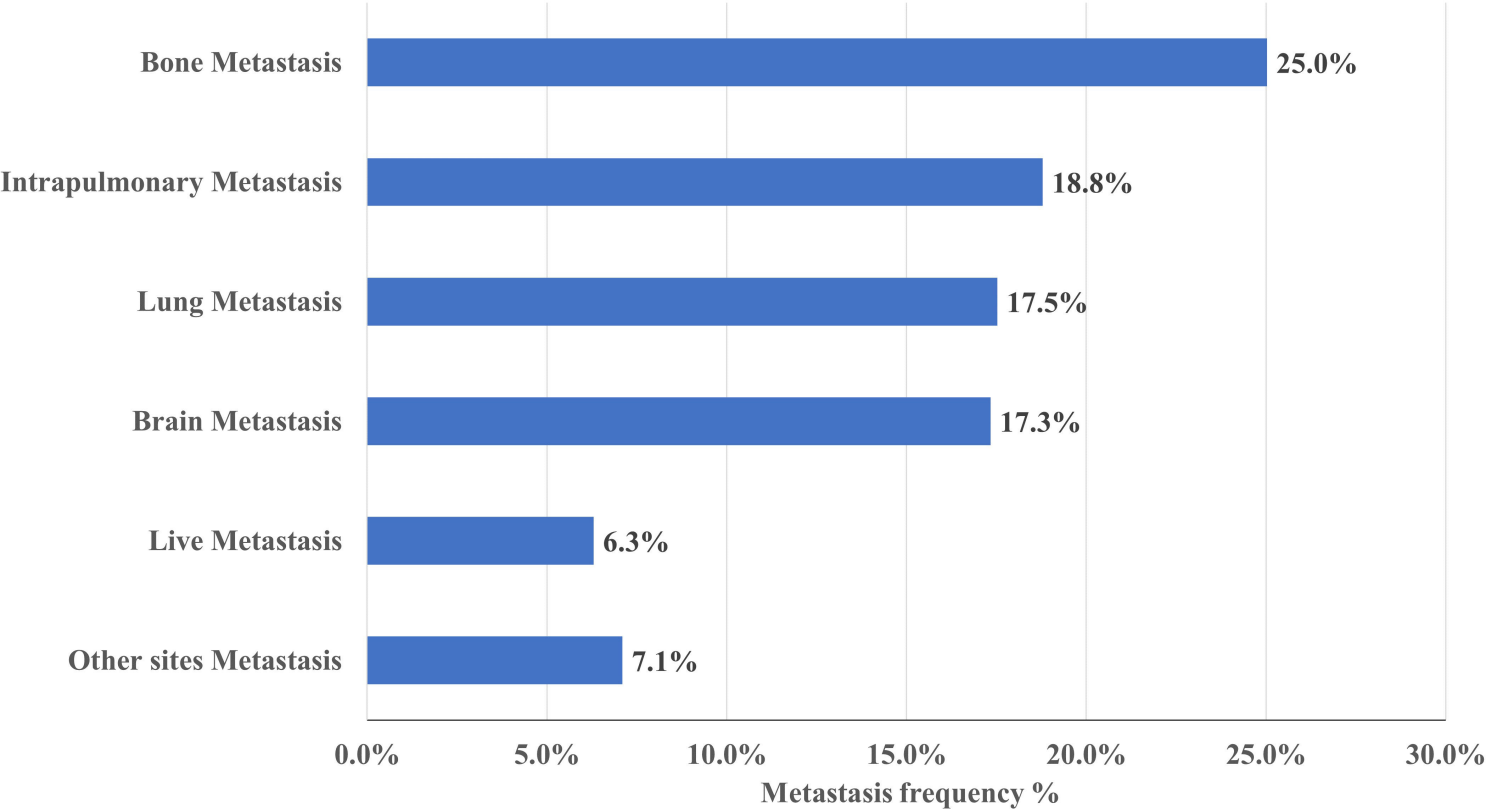
Frequency of metastases in NSCLC. Bone is the most common site of metastasis, occurring in approximately 25% of NSCLC.

As shown in **Figure 1**, among 1586 patients with non-small cell lung cancer, 1198 (75.5%) had genetic mutations detected, and the most common genetic mutation was EGFR (872,55.0%). The frequency of mutations in the other genes were KRAS (120,7.6%), ALK (62,3.9%), HER2 (46,2.9%), RET (24,1.5%), MET (22,1.4%), BRAF (17,1.1%), ROS1(16,1.0%), NRAS (5,0.3%) and PIK3CA (4,0.3%) (**Figure 1**). As shown in **Figure 1**, in patients with the EGFR mutation, the most common type of EGFR mutation was the L858R point mutation (421 cases, 26.5%). The deletion of exon 19 was detected in 367 (23.1%) patients, T790M mutation in 13 (0.8%) patients (19Del+T790M 7 cases, L858R+T790M 5 cases, L858R+G719X+T790M 1 case), and other rare EGFR mutations were detected in 59 (3.7%) patients (exon 20 insertion 29 cases, G719X 19 cases, L861Q 10 cases, S768I 1 case). Two types of EGFR mutations were detected in 12 (0.8%) patients (L858R+19Del 6 cases, G719X+S768I 4 cases, 19Del+20Ins 1 case, and L858R+S768I 1 case) (**Figure 1**). Two genetic mutations were detected in 10 patients. Combined mutations in EGFR and other genes were detected in 6 (0.4%) patients (L858R+MET, L858R+RET, L858R+KRAS, L858R+ PIK3CA, 20Ins+PIK3CA, 19Del+PIK3CA) (**Figure 2**). Additional combinations of genes were detected in four patients (KRAS+PIK3CA, KRAS+ROS1, HER2+PIK3CA, RET+BRAF).

### Metastasis site

As shown in **Figure 2**, of the 1586 NSCLC cases, 796(50.2%) patients had metastasis, 397(25.0%) patients had bone metastasis, 298(18.8%) patients had intrapulmonary metastasis, 278(17.5%) patients had lung metastasis, 275(17.3%) patients had brain metastasis, 100(6.3%) patients had liver metastasis and 112 (7.1%) patients had other metastatic sites.

### Analysis of molecular features with metastatic sites

As shown in **Tables 2**, **3**, 431 (49.1%) patients in the EGFR mutation group developed metastasis, lung metastasis (166 cases, 18.9%, P = 0.004), brain metastasis (166 cases, 18.9%, P = 0.001) and bone metastasis (238 cases, 27.1%, P =0.004)were more common in the EGFR mutation group than in the wild-type group. The EGFR 19Del mutation group had more common lung metastasis (88 cases, 24%, P < 0.001), bone metastasis (94 cases, 25.6%, P = 0.048) and pleural metastasis (85 cases, 23.2%, P = 0.027). The EGFR L858R mutation group was more prone to metastasize to brain (89, 21.1%, P = 0.001) and bone (121 case, 28.7%, P = 0.002). In addition, no difference in the EGFR rare mutation group and 19Del/L858R+T790M mutation group compared with wild-type group metastasis.

**Table 2:** Analysis of the association between metastases and genetic mutations.

genetic mutations	Metastasis			Lung metastasis			Brain metastasis			Bone metastasis			Live metastasis			Intrapulmonary metastasis			other siters metastasis		
	Yes	No	*P* value	Yes	No	*P* value	Yes	No	*P* value	Yes	No	*P* value	Yes	No	*P* value	Yes	No	*P* value	Yes	No	*P* value
**EGFR**	431	447	0.584	166	712	***0.004***	166	712	***0.001***	238	640	***0.004***	47	831	0.553	168	710	0.313	47	831	***0.015***
**ALK**	44	18	***<0.001***	15	47	***0.013***	15	47	***0.007***	20	42	***0.024***	12	50	***0.001***	18	44	***0.021***	8	54	0.334
**ROS1**	10	6	0.237	5	11	***0.045***	3	13	0.42	2	14	0.747	0	16	0.613	6	10	***0.044***	2	14	0.649
**RET**	12	12	0.806	5	19	0.216	3	21	0.751	8	16	0.118	3	21	0.202	6	18	0.275	2	22	1.000
**MET**	14	8	0.139	2	20	1.000	5	17	0.168	7	15	0.175	0	22	0.63	4	18	0.774	3	19	0.445
**KRAS**	65	55	0.197	26	94	***0.012***	28	92	***0.001***	31	89	0.143	9	111	0.61	15	105	0.264	10	110	0.817
**NRAS**	5	0	***0.025***	3	2	***0.017***	0	5	1	2	3	0.259	0	5	1.000	3	2	***0.038***	0	5	1.000
**BRAF**	14	3	***0.005***	5	12	0.057	1	16	0.706	4	13	0.755	0	17	0.613	8	9	***0.005***	3	14	0.207
**PIK3CA**	1	3	0.625	0	4	1.000	0	4	1	0	4	0.324	0	4	1.000	1	3	0.523	0	4	1.000
**HER2**	13	33	***0.014***	2	44	0.107	6	40	0.773	8	38	0.721	4	42	0.522	4	42	0.158	2	44	0.406
**Negative**	184	204		48	340		45	343		76	312		24	364		65	323		35	353	

P values in bold italic reached statistical significance.

**Table 3:** Analysis of the association between metastases and EGFR mutations.

EGFR mutations	Metastasis			Lung metastasis			Brain metastasis			Bone metastasis			Live metastasis			Intrapulmonary metastasis			other siters metastasis		
	Yes	No	*P* value	Yes	No	*P* value	Yes	No	*P* value	Yes	No	*P* value	Yes	No	*P* value	Yes	No	*P* value	Yes	No	*P* value
**19DEL**	187	180	0.332	88	279	***<0.001***	60	307	0.059	94	273	***0.048***	24	343	0.842	85	282	***0.027***	23	344	0.156
**L858R**	203	218	0.821	67	354	0.149	89	332	***<0.001***	121	300	***0.002***	18	403	0.221	70	351	0.962	17	404	***0.004***
**Rare mutations**	25	34	0.469	7	52	0.912	12	47	0.061	14	457	0.460	2	57	0.556	8	51	0.536	5	54	0.891
**19Del/L858R+** **T790M**	9	4	0.122	2	11	0.670	3	10	0.195	4	9	0.302	2	11	0.203	3	10	0.469	0	13	0.615
**Negative**	184	204		48	340		45	343		76	312		24	364		65	323		35	353	

P values in bold italic reached statistical significance.

As shown in **Table 2**, the ALK mutation group (44 cases, 71.0%, P = 0.001) was more prone to metastasis than the wild-type group. Most metastatic sites in the ALK mutation group were lung (15 cases, 24.2%, P = 0.013), brain (15 cases, 24.2%, P = 0.007), bone (20 cases, 32.3%, P = 0.024), liver (12 cases, 19.4%, P = 0.001), and pleura (18 cases, 29.0%, P = 0.021). The BRAF mutation group (14 cases, 82.4%, P = 0.005) was more prone to metastasis than the wild-type group. Pleural metastasis was common in the BRAF mutation group (8 cases, 47.1%, P = 0.005). The NRAS mutation group (5 cases, 100%, P = 0.025) was more prone to metastasis than the wild-type group. Lung metastasis (3 cases, 60.0%, P = 0.017) and plural metastasis (3 cases, 60.0%, P = 0.038) frequently occurred in the NRAS mutation group. In the KRAS mutation group, 120 cases (54.2%) developed tumor metastasis, and the frequent sites were lung metastasis (26 cases, 21.7%, P = 0.012) and brain metastasis (27 cases, 23.3%, P = 0.001). In the ROS1 mutation group, 16 patients (62.5%) developed tumor metastasis, and lung metastasis (5 cases, 31.3%, P = 0.045) and pleural metastasis (6 cases, 37.5%, P = 0.044) often occurred. Compared with the wild-type group, the HER2 mutation group had less metastasis (13 cases, 28.3%, P = 0.014). In addition, there was no difference in the RET mutant group, MET mutant group and PIK3CA mutant group compared with wild-type group metastasis.

## Discussion

This study demonstrates different results for common metastatic sites in NSCLC patients with common genetic mutations. First, as EGFR was the most common mutation in this study, we studied the common sites of metastasis in 872 NSCLC patients with EGFR mutations. In terms of single organ metastasis, EGFR mutant NSCLC prefer to transfer to bone, brain, and lung, with L858R mutation patients mostly developing bone and brain metastasis, 19Del mutation patients mostly developing intrapulmonary, pleural, and bone metastasis. This is in consistent with previous studies on EGFR mutant NSCLC metastasis. Although a retrospective study found no metastasis site differences in EGFR mutation versus wild-type NSCLC patients. However, this study has only involved 105 patients, and there may be a bias (20). The study of Chen et al. showed that EGFR 19Del is usually associated with intrapulmonary and bone metastasis, with higher metastasis rate of bone, brain, and liver in patients with concurrent T790M mutation (21). Kuijpers et al. showed that the classical EGFR mutation (19Del/L858R) was associated with pleural and bone metastasis (22). These results are generally in agreement with our study, but we did not find differences in metastatic sites in patients with coexisting T790M mutation and wild-type patients. In addition, EGFR mutation showed no association with liver metastasis in NSCLC patients, which is consistent with studies in the literature (23).

ALK rearrangement is one of the most common targeted mutations in NSCLC, ALK mutations in 3.9% of NSCLC were detected in this study, metastasis in 71% of ALK positive NSCLC patients, the metastasis rate was much higher than that in the wild-type group (71.0% vs. 47.4%, P < 0.001). The most common site of metastasis was bone (32.3% vs. 19.6%, P = 0.024), followed by the pleura (29% vs. 16.8%, P = 0.021), lung (24.2% vs. 12.4%, P = 0.013), brain (24.2% vs. 11.6%, P = 0.007) and the liver (19.4% vs. 6.2%, P = 0.001). In previous studies, ALK positive NSCLC often developed metastasis, with brain metastasis being the most common, with a baseline cohort metastasis rate of approximately 24% – 29%, followed by bone metastasis of approximately 27% (24–26). However, the results of pleural metastasis and lung metastasis are inconsistent, and ALK+NSCLC Meta-analysis by Dexter et al. showed that patients with ALK rearrangement NSCLC are more likely to develop pleural metastasis, but are less likely to develop lung metastasis (12). However, the study of Gang Chen et al. showed that in 97 ALK-positive NSCLC, the incidence of brain metastases was significantly higher in ALK-positive patients both at baseline and during treatment than in the negative group, but the pleural effusion was opposite (25). Di Ma et al. analyzed 52 patients with ALK-positive advanced NSCLC at the Cancer Hospital of Chinese Academy of Medical Sciences, and the incidence of pleural metastasis was 36.5% (27). In the review by Chang et al., metastases of central nervous system (CNS), Bone, pleura, and liver often occurred in ALK positive NSCLC, which was basically consistent with our study (28). Moreover, mechanistic studies of metastasis in ALK-positive NSCLC were unclear. A novel circular RNA F-circEA-2a mainly located in the cytoplasm generated by the EML4-ALK fusion gene was found, and although F-circEA-2a does not affect the proliferation of NSCLC cells, it can significantly affect the migration and invasion of cancer cells (29). In addition, epithelial-mesenchymal transformation (epithelial-mesenchymal transition, EMT) is closely associated with cancer cell invasion and metastasis, and EML 4-ALK expression also increases the expression of EMT-inducible transcription factors, snail and slug, indicating that EMT is ongoing (30). All these studies showed that EML 4-ALK fusion is associated with invasion and metastasis of NSCLC.

ROS1 are also important drivers of NSCLC and are found in 1-2% of NSCLC patients (28, 31, 32). In our study, the ROS1 mutation rate was 1.0% (16/1586), in single organ metastasis, ROS1-positive NSCLC was more transferred to the pleura (37.5% vs.16.8%, P = 0.044) and lung (31.3% vs.12.4%, P = 0.045) compared with wild type. The brain metastasis rate of ROS 1-positive NSCLC was 18.75% (3/16, P = 0.42), but there was no statistical difference due to the small number of cases. The largest prospective trial of ROS1 TKI in East Asia (including Taiwan, Japan, Korea, China) reported a CNS transfer rate of 18.1% in ROS 1-positive NSCLC at baseline, which is consistent with our study (33). Jun et al. found that the ROS 1 cells expressing the CD74-ROS 1 fusion were highly invasive *in vitro* and metastatic *in vivo*. Expression of CD74-ROS 1 results in the phosphorylation of the extended synaptotagin-like protein E-Syt 1 (34). Another study further confirmed cellular EMT in CD74-ROS 1 or CD74-ROS1 G2032R mutation by constructing a G2032R mutant A549-CD74 ROS1 crizotinib resistant cell line. This promotes migration and increases the expression of matrix metalloproteinase (MMP) -9 and Twist1 transcription factors (35). In a study from Shanghai Chest Hospital, showing that the specific ROS 1 fusion variant CD74-ROS 1 may increase the preference for CNS metastasis, among 19 CD74-ROS1 patients, 31.6% had CNS metastases compare to no (0%) CNS metastasis among the 17 non-CD74-ROS1 patients (36).

KRAS is an important driver gene of NSCLC, the KRAS mutation rate in the Chinese NSCLC population is approximately 12.1% (37). Studies of KRAS and NSLCL metastasis are rare, and the results are inconsistent. As Chan KH et al. showed that in single-organ metastasis, KRAS/NRAS mutated NSCLC tended to transfer to the brain and bone, however, the sample size of this study was relatively small, with only 29 cases of KRAS/NRAS mutated NSCLC (38). However, KRAS was not considered to be associated with distant metastasis of NSCLC in a meta-analysis study by Wu Y et al. (15). In this study, KRAS mutation rate was 7.6% (120/1586), and in single organ metastasis, KRAS mutant NSCLC tended to transfer to brain (23.3% vs.11.6%, P = 0.001) and lung (21.7% vs.12.4%, P = 0.012) compared with wild type. The study population of Chan KH et al. was western group, and the population included in Wu Y et al. et al. meta-analysis was admixed population containing 10249 Western patients and 26319 Asian patients, while our study was single-center Chinese population, which may have ethnic and geographical differences, leading to incomplete consistent study results.

In this study, the frequency of HER 2 mutations in NSCLC was approximately 2.9% (46/1586), which is consistent with previous reports. This study showed that patients with HER2-mutated NSCLC had less metastasis compared with wild type (28.3% vs. 47.4%, P = 0.014), however, Le Y reported that HER2 positivity was associated with interpulmonary metastasis, but the intrapulmonary metastasis rate in patients with HER2 mutant NSCLC was only 4.3% in this study (39).

In addition, we also analyzed the association of rare mutations such as BRAF, NRAS, RET, MET, and PIK3CA with metastasis in NSCLC patients. When stratified by mutation profiles, patients with BRAF mutation or NRAS mutation NSCLC were more susceptible to metastasis (BRAF, 82.4% vs. 47.4%, P = 0.005; NRAS, 100% vs. 47.4%, P = 0.025). Among these, NSCLC patients with BRAF mutations had more pleural metastasis (47.1% vs.16.8%, P = 0.005), and NRAS mutations had lung (60.0% vs.12.4%, P = 0.025) and pleural metastasis (60.0% vs.16.8%, P = 0.038). However, a negative association between BRAF and distant metastasis of NSCLC was previously reported by Wu Y et al. (15). The study of Chan KH et al. showed that NRAS mutant NSCLC preferred to transfer to the brain and bone (38). In addition, in terms of total metastasis rate and single organ metastasis, the RET mutation, MET mutation and PIK3CA mutation groups were not different compared with the wild-type group, and the sample size of the cohort with rare mutation was small, which makes the analysis and interpretation difficult.

This study has several strengths. First, this is a comprehensive study to explore the correlation between NSCLC gene profile and metastasis. We not only investigated the relationship of common gene mutations such as EGFR, ALK, ROS1 and NSCLC metastasis, but also extended the gene spectrum to rare gene mutations such as KRAS, HER2, BRAF, RET, MET, and PIK3CA. Secondly, the large number of NSCLC patients included in this study makes the study results more credible. To the best of our knowledge, this is the first study of the correlation between gene mutation profile and metastasis in Chinese NSCLC patients based on real-world data.

However, this study also has some limitations. First, this was a single-center retrospective study, which may limit the generalizability of the results. Second, due to the low rate of NSCLC rare gene mutations, the small sample size of our rare gene mutation cohort makes analysis and interpretation difficult. However, the mechanism of different gene transfer preferences was unclear and further studies were essential, which may be necessary to find new ways to limit the development of metastasis.

## Conclusions

This retrospective study further suggested a correlation between gene profiles and metastatic sites in NSCLC patients. First, ALK mutations, BRAF mutations, and NRAS mutations were all positively associated with NSCLC metastasis, while HER2 mutations in NSCLC had less metastasis. Secondly, in terms of single organ metastasis, EGFR mutation was associated with lung, brain, and bone metastasis of NSCLC, KRAS mutation was associated with lung and brain metastasis of NSCLC, and ROS1 mutation was associated with lung and pleural metastasis of NSCLC, but the total metastasis rate was not different compared with wild type. Furthermore, no association was found between RET, MET, PIK3CA mutations and NSCLC metastasis. This is an important study using real-world data to predict metastasis in NSCLC patients, and these results need to be confirmed in larger studies.

## Data Availability

The original contributions presented in the study are included in the article/**Supplementary Material**. Further inquiries can be directed to the corresponding author.
